# MicroRNA-122 ameliorates corneal allograft rejection through the downregulation of its target CPEB1

**DOI:** 10.1038/cddiscovery.2017.21

**Published:** 2017-05-15

**Authors:** Ting Wang, Fengjie Li, Wenwen Geng, Qingguo Ruan, Weiyun Shi

**Affiliations:** 1 State Key Laboratory Cultivation Base, Shandong Provincial Key Laboratory of Ophthalmology, Shandong Eye Institute, Shandong Academy of Medical Sciences, Qingdao, People's Republic of China; 2 Institute of Biomedicine and Biotechnology, Shenzhen Institutes of Advanced Technology, Chinese Academy of Sciences, Shenzhen, People's Republic of China

## Abstract

Transplant rejection is a major cause of corneal transplantation failure. MicroRNAs (miRNAs) are a family of small RNAs that regulates gene expression in a sequence-specific manner. miRNAs have recently been shown to have important roles in human organ transplantation, but reports of miRNAs directly associated with corneal transplantation rejection remain limited. To investigate the role of miRNAs during corneal allograft rejection, we established a mouse penetrating keratoplasty model and used microarrays to screen for differentially expressed miRNAs. Our results revealed that the expression of miR-122 was significantly decreased in the allogeneic group. Consistent with this result, the expression of cytoplasmic polyadenylation element-binding protein-1 (CPEB1), a direct target of miR-122, was significantly increased. Further analysis demonstrated that miR-122 inhibited inflammatory cytokine-induced apoptosis in corneal keratocytes through the downregulation of its target CPEB1. We also found that increased miR-122 expression significantly reduced the risk of corneal transplantation rejection. Thus, our results indicate that miR-122 is an important miRNA associated with corneal graft rejection and can be used as a therapeutic target for the prevention of immune rejection after keratoplasty.

## Introduction

Organ transplantation is the optimal therapeutic intervention in patients with end-stage organ failure. According to a survey by the World Health Organization, there are approximately 60 million corneal blindness patients worldwide.^1–3^ Corneal transplantation is the major method for eyesight restoration;^1^ however, transplant rejection is the major reason for surgical failure.^4–7^ The cumulative incidence rate of rejection occurrence 10 years after keratoplasty is >20%, and the incidence of immune rejection after corneal transplantation in high-risk patients with severe infection or chemical injury reaches 60–90%.^8,9^ Many factors, including inflammation and cell apoptosis, contribute to corneal transplantation rejection.^10^ Immunosuppressants are usually used in clinical practice to reduce the risk of postoperative rejection.^11^ However, long-term use of immunosuppressants can cause side effects, including inhibition of immune functions and adverse drug reactions.

MicroRNAs (miRNAs or miRs) are a group of non-coding single-stranded RNA molecules that are approximately 22 nucleotides in length. They can bind to the mRNAs of their target genes in a sequence-specific manner, resulting in degradation of the mRNA or inhibition of translation; therefore, miRNAs have important roles in biological development, cell differentiation, cell apoptosis and tumor development.^12–14^ Although studies have confirmed the roles of miRNAs in human organ transplantation,^11–13^ there are few reports on miRNAs directly associated with corneal transplantation rejection. miR-466 and miR-184 are reported to be closely related with corneal lymphangiogenesis.^15,16^ Studies have also shown that miR-132, miR-184 and miR-204 are associated with corneal neovascularization.^15,16^ Corneal lymphangiogenesis and corneal neovascularization are important risk factors for the development of corneal allograft rejection.^17^ However, the mechanism of the involvement of miRNAs in allograft rejection is not clear.

Using miRNA expression profile analysis, this study showed that miR-122 is an important miRNA that was negatively correlated with corneal transplantation rejection. Further investigations demonstrated that miR-122 blocked apoptosis in corneal keratocytes and thus reduced the risk of immune rejection after keratoplasty through the downregulation of its target, cytoplasmic polyadenylation element-binding protein-1 (CPEB1).

## Results 

### miR-122 is an important miRNA associated with corneal transplantation rejection

**Figure 1:**
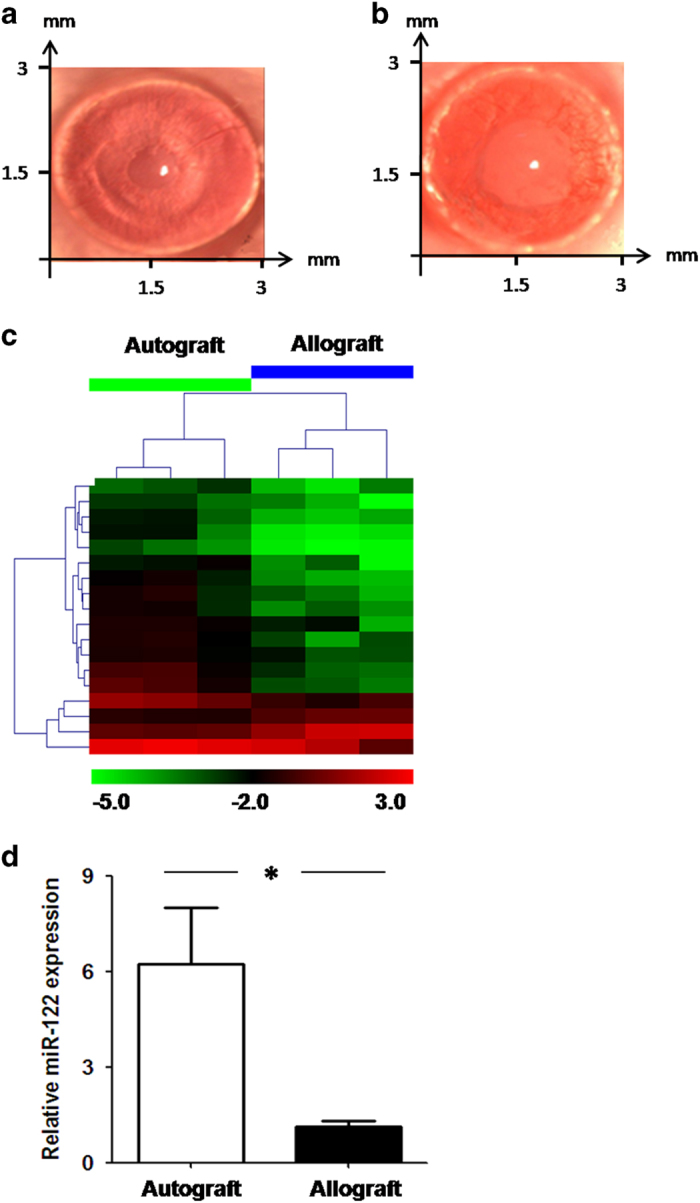
Identification of miRNAs associated with corneal transplantation rejection. (**a** and **b**) Morphology of the mouse cornea 14 days after corneal transplantation: (**a**) the autograft group; (**b**) the allograft group. (**c**) Cluster analysis of the miRNA expression profile in the transplanted cornea with (allograft) or without rejection (autograft). (**d**) Downregulation of miR-122 in the allograft group was confirmed by real-time PCR. The results are representative of three independent experiments. *P<0.05.

To screen for miRNAs associated with immune rejection after corneal transplantation, we established a mouse PKP model. Three days after the corneal allograft (BALB/s mice received corneal grafts from C57BL/6 mice), the corneal grafts exhibited mild edema, visible pupil and iris vessels, and new blood vessels in the limbus and edge of the implant bed. Seven days post-surgery, the growth of new blood vessels reached the peripheries of the suture. Ten to 14 days post-surgery, the corneal grafts had significant edema and turbidity, the anterior chamber and iris were not visible, and a large number of new blood vessels had grown into the grafts (Figures 1a and b).

We used the miRCURY LNA Array platform to evaluate the differentially expressed miRNA profiles in mice 14 days after PKP. miRNAs with differences in expression ≥1.5-fold and *P*-values ≤0.05 were selected for the volcano plot (Figure 1c). Eight miRNAs exhibited significant expression differences, including two that were significantly upregulated and six that were significantly downregulated in the allograft group (Table 1).

Three miRNA target prediction programs (TargetScan, miRanda and PicTar) were used to screen for possible target genes. CPEB1, an important mediator of cell ageing and apoptosis,^18–20^ was identified as the target gene of both miR-122 and miR-1a, two miRNAs that were downregulated in the allograft group. In addition, miR-122 exhibited the largest expression difference among all eight miRNAs identified. Therefore, miR-122 and CPEB1 were chosen for further study. The microarray result for miR-122 (Figure 1d) was further validated by real-time PCR.

### CPEB1 was a target of miR-122

**Figure 2:**
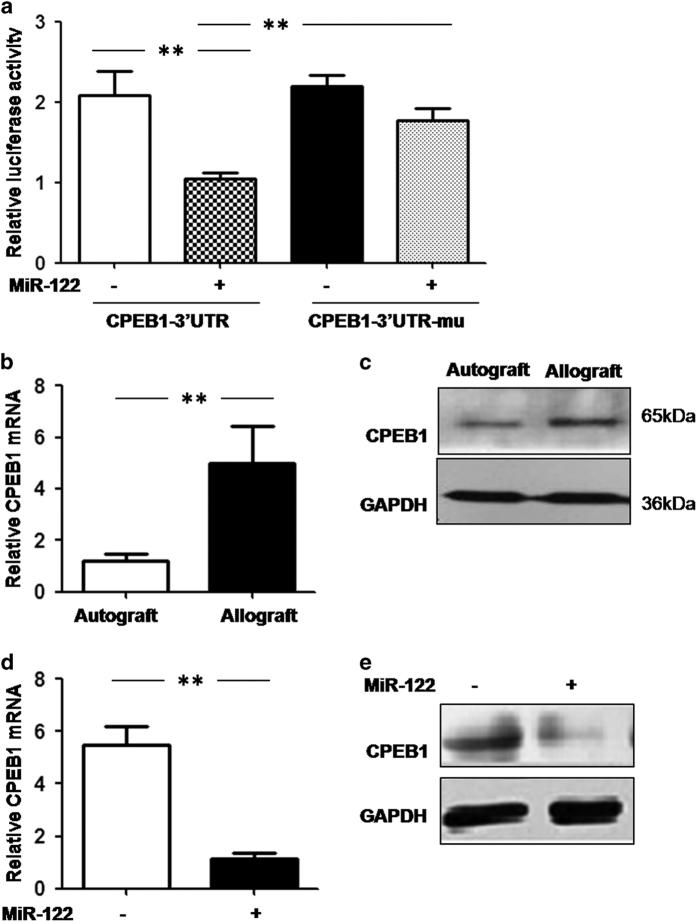
CPEB1 is a direct target of miR-122. (**a**) Luciferase reporter plasmids carrying the wild-type (CPEB1-3′-UTR) or miR-122-binding site-mutated CPEB1 3′-UTR (CPEB1-3′-UTR-Mu) were transfected into a TKE2 corneal endothelial cell line with or without miR-122 overexpression. After 24 h, the luciferase activities of the total cell lysates were measured using a dual-luciferase reporter assay. Co-transfection of the Renilla luciferase expression vector pRL-TK was used as an internal control. (**b** and **c**) CPEB1 mRNA (**b**) and protein (**c**) expression levels were examined in the autograft and allograft group after corneal transplantation by RT-PCR and western blotting, respectively. (**d** and **e**) TKE2 corneal endothelial cell lines were transfected with agomir-122, and CPEB1 mRNA (**d**) and protein (**e**) expression levels were examined as in **b** and **c**. The results are representative of three independent experiments. **P<0.01.

To validate CPEB1 as a target of miR-122, CPEB1 3′-untranslated regions (UTRs) with or without mutations in the miR-122-binding site were inserted into the pmiR-RB-REPORT dual-luciferase reporter plasmid. Our dual-luciferase reporter assay showed that miR-122 overexpression significantly downregulated the fluorescence of the reporter plasmid containing the wild-type CPEB1 3′-UTR but not the CPEB1 3′-UTR with mutations in the miR-122-binding sites (Figure 2a).

To further confirm that miR-122 regulates CPEB1 expression, we performed real-time PCR and western blotting to compare CPEB1 expression in the corneal autograft and allograft groups. Our results showed that CPEB1 mRNA (Figure 2b) and protein (Figure 2c) expression levels were significantly increased in the corneal autograft group. We also overexpressed miR-122 in the TKE2 mouse corneal epithelial cell line and measured CPEB1 expression using real-time PCR and western blotting. miR-122 overexpression significantly downregulated CPEB1 mRNA (Figure 2d) and protein (Figure 2e) expression compared with the control group. Taken together, these results indicate that CPEB1 was a target of miR-122 in the corneal cells.

### Expression of miR-122 is enriched in corneal stromal cells

**Figure 3:**
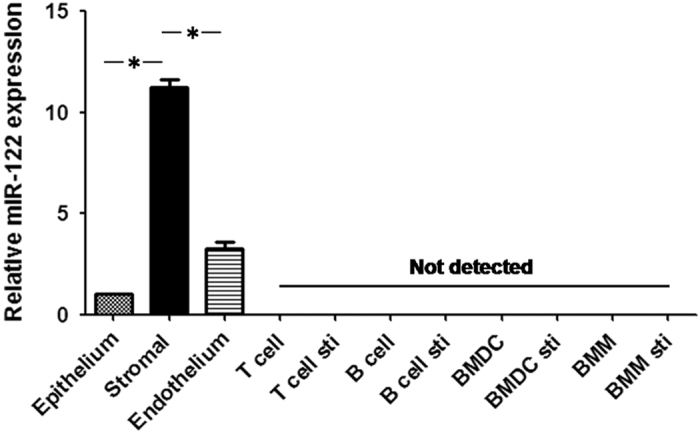
Expression of miR-122 is abundant in corneal stromal cells as well as in the liver. Total RNA was extracted from mouse corneal cells (epithelia, stroma and endothelia) and different types of immune cells (T-cell, B-cell, dendritic cells and macrophages) with or without stimulation, as stated in the Materials and methods section. The mRNA expression level of miR-122 was detected using real-time PCR. The results are representative of three independent experiments. *P<0.05. BMDCs, bone marrow-derived dendritic cells; BMMs, bone marrow-derived macrophages; Sti, stimulated.

To elucidate the mechanism underlying the role of miR-122 in corneal transplantation rejection, we evaluated the expression of miR-122 in three types of corneal cells and four types of immune cells with or without stimulation. Our results revealed high expression of miRNA-122 in the corneal stromal cells but no expression in immune cells (Figure 3). Therefore, the subsequent study focused on the effects of miR-122 on the biology of corneal keratocytes.

### miR-122 blocks apoptosis in a mouse corneal keratocyte cell line through the downregulation of CPEB1

**Figure 4:**
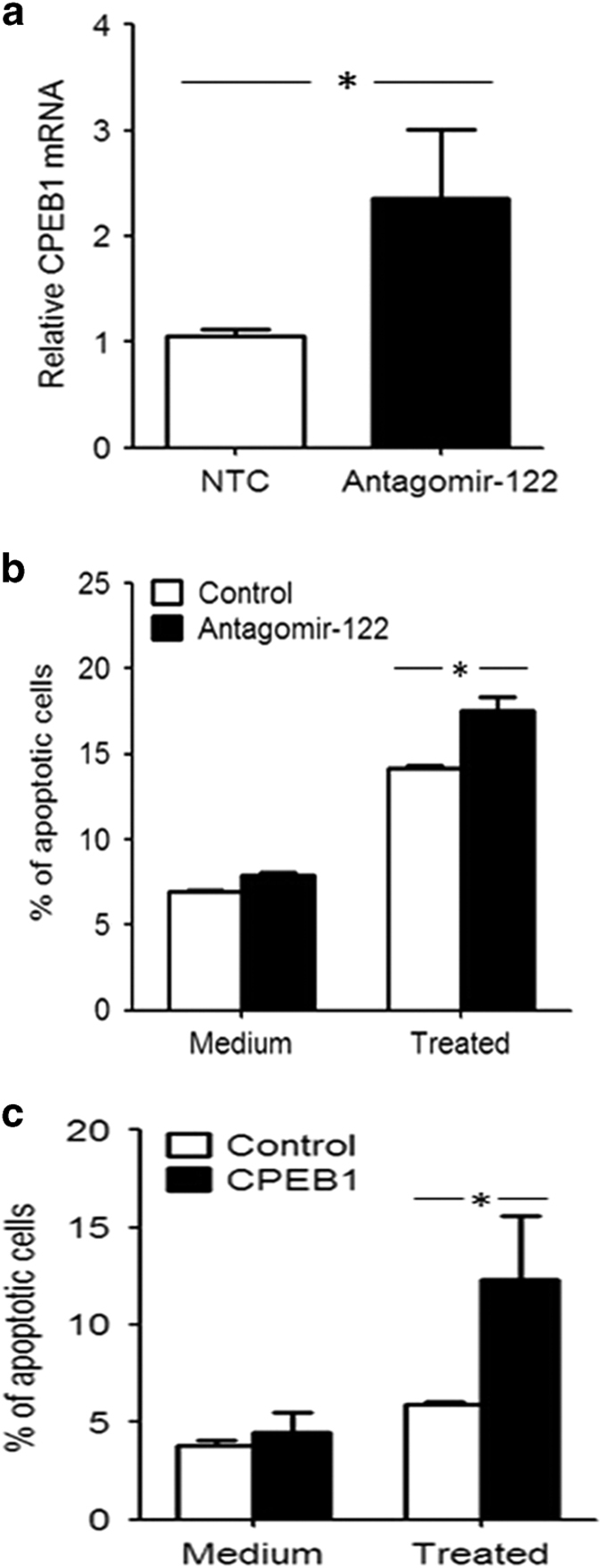
miR-122 inhibits whereas CPEB1 promotes inflammatory cytokine-induced apoptosis of a corneal keratocyte cell line. (**a** and **b**) The corneal keratocyte cell line was transfected with the agomir negative control (control) or antagomir-122 and treated with or without cytokines (100 ng/ml TNF-*α*, IL-1*β* and TNF-*γ*). The mRNA expression of CPEB1 (**a**) was examined by real-time PCR. Apoptosis (**b**) was detected after 48 h by staining cells with annexin V and analysis by flow cytometry. (**c**) The corneal keratocyte cell line was transfected with a control or CPEB1 overexpression plasmid (CPEB1) and treated with or without cytokines (100 ng/ml TNF-*α*, IL-1*β* and TNF-*γ*). Apoptosis was detected as in **b**. The results are representative of three independent experiments. *P<0.05.

To examine the effects of miR-122 on apoptosis in corneal keratocytes, we suppressed miR-122 expression using antagomir-122 and treated the cells with inflammatory cytokines. Inhibition of miR-122 expression increased CPEB1 expression (Figure 4a) and promoted cytokine-induced apoptosis in the corneal keratocytes (Figure 4b) compared with the control group.

To detect the effects of CPEB1 on apoptosis in the corneal keratocytes, a CPEB1 overexpression plasmid was transfected into the corneal keratocyte cell line, which was then treated with or without inflammatory cytokines. CPEB1 overexpression promoted cytokine-induced apoptosis in the corneal keratocytes (Figure 4c).

**Figure 5:**
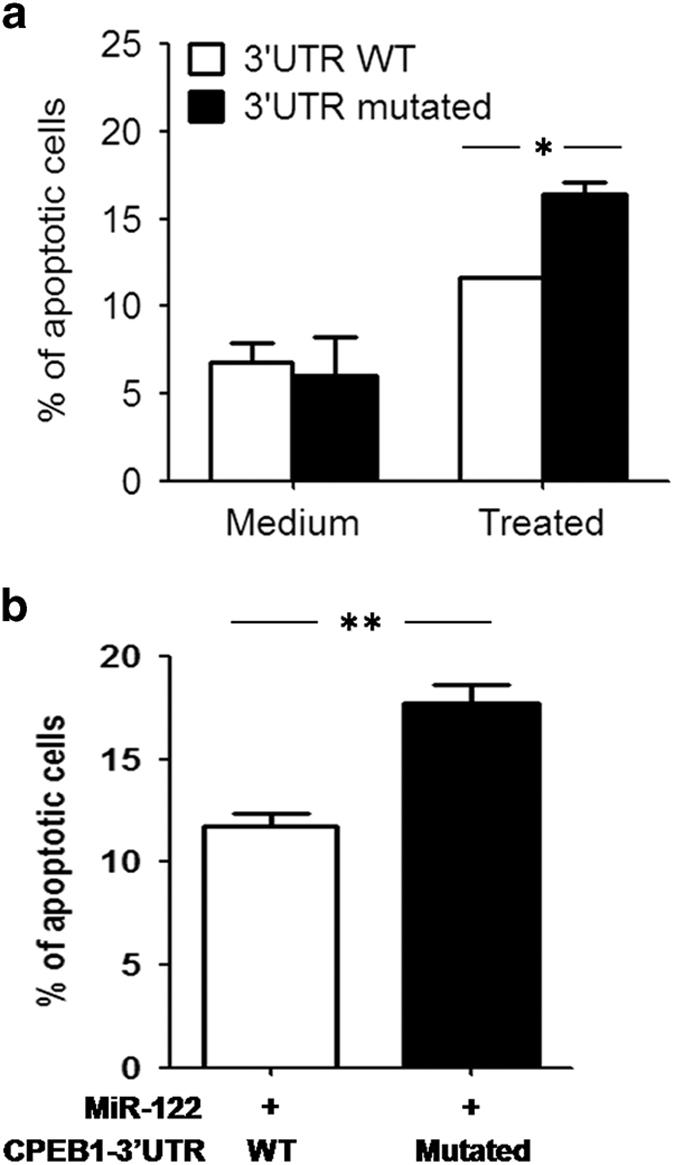
miR-122 inhibits the apoptosis of corneal keratocytes through the downregulation of CPEB1. (**a**) The corneal keratocyte cell line was transfected with CPEB1 overexpression plasmids carrying either the wild-type (3′-UTR WT) or miR-122-binding site-mutated 3′-UTR (3′-UTR mutated) and stimulated with or without cytokines (100 ng/ml TNF-*α*, IL-1*β* and TNF-*γ*). The percentage of apoptotic cells (annexin V+) was determined after 48 h by staining cells with annexin V and analysis by flow cytometry. (**b**) The corneal keratocyte cell line was treated as in **a**, and agomir-122 was used to overexpress miR-122. Apoptosis was detected after 48 h as in **a**. The results are representative of three independent experiments. *P<0.05, **P<0.01.

To determine whether miR-122 inhibited apoptosis in the corneal keratocytes through CPEB1 downregulation, we treated the corneal keratocyte cell line with inflammatory cytokines and transfected a CPEB1 overexpression plasmid carrying either a wild-type (CPEB1-WT) or miR-122-binding site-mutated (CPEB1-Mu) 3′-UTR. Keratocyte apoptosis was significantly increased in the CPEB1-Mu group compared with the CPEB1-WT group (Figure 5a). Similar results were obtained after miR-122 overexpression (Figure 5b). These results indicate that miR-122 inhibited apoptosis in corneal keratocytes through the downregulation of CPEB1.

### Increased miR-122 expression significantly reduces the risk of corneal transplantation rejection

**Figure 6:**
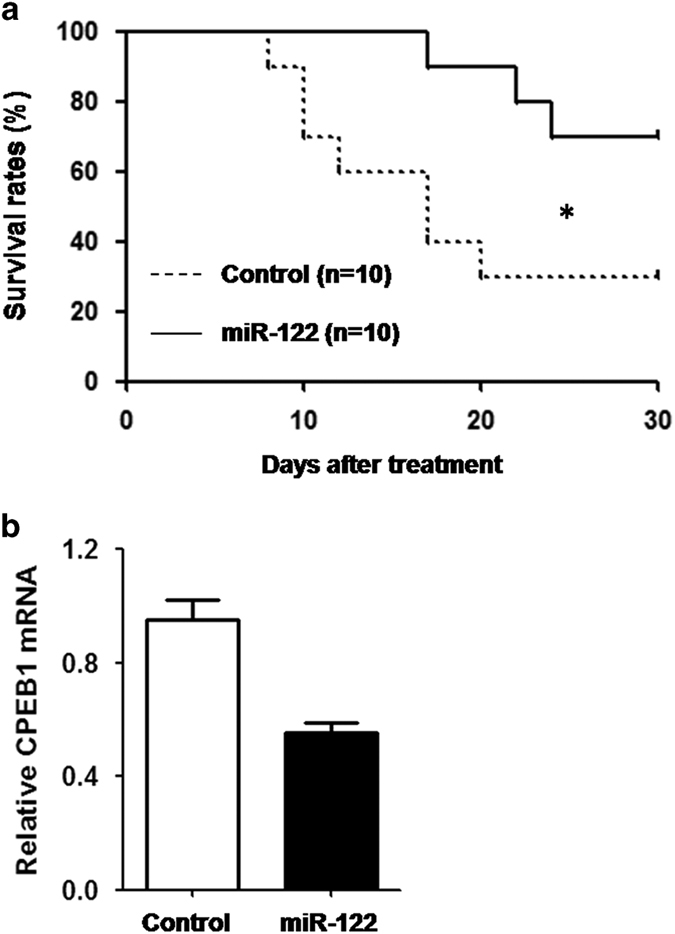
Increased miR-122 expression significantly decreases the risk of corneal transplantation rejection. The mouse PKP model was established according to the procedures in the Materials and methods section. The mice were locally treated with agomir-negative control (control) or agomir-122 (miR-122) in the eye. (**a**) The survival conditions of the grafts in the two groups were observed daily for 30 days post-treatment. (**b**) mRNA expression of CPEB1 was examined by real-time PCR. The results are representative of two independent experiments. *P<0.05.

To investigate whether increased miR-122 expression can reduce corneal cell apoptosis and thus decrease the risk of corneal transplantation rejection, we established a mouse PKP model and treated mice with either the agomir negative control or agomir-122 (to increase the expression of miR-122). The grafts in the mice in these two groups were observed for 30 days after treatment. The survival rate of the mouse grafts was significantly increased in the experimental group that received local administration of agomir-122 compared with the control group (Figure 6a). Consistent with this phenotype result, CPEB1 expression was also significantly decreased in the agomir-122-treated group (Figure 6b).

## Discussion

Corneal transplantation is an important treatment for the restoration of eyesight in corneal blindness patients. Although studies of the mechanism underlying rejection have achieved some progress, the incidence of rejection after keratoplasty remains high. Thus, other unrecognized pathways may underlie postoperative rejection.

miRNAs are a large group of small, non-coding RNAs that control the expression of target genes at the posttranscriptional level. More than 200 miRNA species are expressed in the eye, of which 25% are found in the cornea.^21^ Current studies in ophthalmology have focused on the role of miRNAs in retina- and lens-related diseases.^22,23^ In addition, miRNAs are also involved in the regulation of pathological processes in the cornea, such as keratoconus, corneal neovascularization caused by corneal transplantation, herpes simplex virus infection and alkali burns.^24–27^ However, relevant investigations of the role of miRNAs in corneal transplantation rejection are lacking. Our current study revealed that miR-122 expression was significantly downregulated during rejection after keratoplasty. However, the downregulation of miR-122 may only be correlated with such rejection. Alternatively, miR-122 may directly regulate corneal transplantation rejection. miR-122 is considered a liver-specific miRNA;^28–32^ its expression level accounts for >70% of all miRNAs in the liver. miR-122 has important regulatory roles in physiological functions of the liver, including the growth cycle and lipid metabolism of hepatocytes. The role of miR-122 in corneal cells has not been reported.

To investigate the mechanism of miR-122 in corneal transplantation rejection, we first performed bioinformatics analyses and cross-predictions to identify CPEB1 as a miR-122 target in corneal cells. CPEB1 has been reported to be a target gene of miR-122 in skin fibroblasts. CPEB1 binds to the 3′-UTR of the p53 gene and recruits germline development 2 to promote polyadenylation and thus the translation of the tumor-suppressor p53, thereby promoting the ageing of human fibroblasts.^33–35^ In addition, CPEB1 may regulate cell growth through the regulation of cyclin B1 synthesis.^36^

Many immune cells, including lymphocytes, dendritic cells and macrophages, are present in the cornea after PKP. To confirm the involvement of miR-122 in postoperative rejection in different cell types, we examined miR-122 expression in a variety of immune cells and three layers of mouse corneal tissue (the epithelial, stromal and endothelial layers). miR-122 was highly expressed in the corneal stromal layer and was not expressed in immune cells. Therefore, we next focused on corneal keratocytes to study the functions and mechanisms of miR-122 in corneal transplantation rejection.

During the immune rejection of corneas, immune cells secrete inflammatory cytokines that damage the corneal endothelial, stromal and epithelial layers.^37,38^ Studies have shown that treating corneal stromal cells with inflammatory cytokines causes stromal cell apoptosis and promotes the development of corneal transplantation rejection.^39^ As CPEB1 has important roles in cell ageing and apoptosis,^18–20^ we investigated whether miR-122 regulates corneal stromal cell apoptosis through the reduction of CPEB1 expression. Our results revealed that the inhibition of miR-122 expression or increased CPEB1 expression in a corneal keratocyte cell line significantly increased apoptosis induced by inflammatory cytokines. We also confirmed that miR-122 suppressed corneal keratocyte apoptosis through downregulation of CPEB1. Furthermore, local overexpression of miR-122 in the eye, which was accompanied by decreased expression of CPEB1, significantly increased the survival of the mouse grafts compared with the control group.

The inflammatory environment in the eyes after corneal transplantation is the major factor promoting the early and rapid development of rejection. Although our results showed that miR-122 inhibited apoptosis in corneal stromal cells and significantly decreased the risk of corneal transplantation rejection by reducing the expression of its target CPEB1, whether miR-122 exerts its function only through CPEB1 and the pathways by which CPEB1 regulates corneal stromal cell apoptosis remain unknown. Nevertheless, our results indicate that miR-122 is closely associated with corneal transplantation rejection and may have important roles in this process. Targeting miRNAs may become a new strategy for reducing the risks of rejection after keratoplasty in clinical practice.

## Materials and methods

### Mice

Male BALB/c mice and C57BL/6 mice aged 6–8 weeks with a body weight of 18–23 g were purchased from Beijing HFK Bioscience CO., LTD (Beijing, China). Mice were housed under pathogen-free conditions at the animal facility of Shandong Eye Institute. All procedures were preapproved by the Institutional Animal Care and Use Committee, and all animals were treated according to the Association for Research in Vision and Ophthalmology Statement for the Use of Animals in Ophthalmic and Vision Research.

### Establishment of the mouse PKP model

The mice were divided into two groups. In the autograft penetrating keratoplasty (PKP) group, BALB/c mice underwent ipsilateral rotational autokeratoplasty. In the allograft PKP group, the recipients were BALB/s mice, and the donors were C57BL/6 mice. The diameter of the corneal graft was 2 mm. During manipulation, the endothelium was protected, the graft was on the recipient bed, eight stitches of intermittent suture were performed using 11/0 nylon suture, and sterile air was injected using a no. 32 needle to form an anterior chamber for prevention of injury to the surgically treated eyes. The eyelids were sutured with one intermittent suture stitch using 10/0 nylon suture. After surgery, ofloxacin eye ointment was used once daily, and atropine eye ointment was administered based on the reaction of the anterior chamber. The eyelid suture was removed after 3 days, and the corneal suture was removed after 9 days. The condition of the grafts was observed and recorded daily. The detailed classification standards and scoring indicators were as follows: 0 point, clear corneal graft; 1 point, minimal superficial non-stromal opacity and a visible pupil margin and iris vessels through the cornea; 2 points, mild deep stromal opacity with a visible pupil margin and iris vessels; 3 points, moderate stromal opacity and a visible pupil margin only; 4 points, intense stromal opacity with some pupil margin visible; and 5 points, maximal corneal opacity with total obscuration of the anterior chamber. A graft score >2 points after keratoplasty indicated graft dysfunction (turbid).

### Microarrays and bioinformatics analysis

Fourteen days post-surgery, the mouse cornea was removed from along the corneal limbus. RNA was extracted and subjected to denatured agarose gel electrophoresis. Image scanning was performed using the Genepix 4000B at 635 nm excitation (AXON, Union City, CA, USA). The data were analyzed using Genepix Pro 6.0 (Axon Instruments, Union City, CA, USA). Three miRNA target prediction programs (TargetScan, miRanda and PicTar) were used to screen for possible target genes.

### Dual-luciferase reporter assay

The 3′-UTR sequence of the CPEB1 gene was amplified using genomic DNA from 3T3 cells as the template and cloned into the pmiR-RB-REPORT dual-luciferase reporter plasmid (KangChen Bio-tech, Shanghai, China). Site-directed mutagenesis of the miR-122-binding site was performed using the QuickChange kit (Stratagene, Santa Clara, CA, USA) according to the manufacturer’s instructions. Mouse full-length CPEB1 cDNA with a wild-type or miR-122-binding site-mutated 3′-UTR was cloned into the pZSGreen lentiviral vector. TKE2 (mouse corneal epithelial progenitor cell line) cells were transiently transfected with constructs containing wild-type or miR-122-binding site-mutated 3′-UTR together with or without miR-122 overexpression using Lipofectamine LTX transfection reagent (Invitrogen, Grand Island, NY, USA). After 24 h, the luciferase activities of total cell lysates were measured using the dual-luciferase reporter assay system (Promega, Madison, WI, USA). Co-transfection of the Renilla luciferase expression vector pRL-TK (Promega) was used as an internal control for all reporter assays.

### Isolation of immune cells

T cells and B cells were isolated from the spleens of normal C57BL/6 mice using an EasySep mouse T-cell or B-cell isolation kit (Stemcell, Vancouver, BC, Canada), respectively. Macrophages and dendritic cells were generated from femoral and tibial bone marrow cells of normal C57BL/6 mice as previously described.^40,41^

### Real-time PCR analysis

Total RNA was extracted using TRIzol lysis buffer (Invitrogen Life Technologies, Grand Island, NY, USA) from the following tissues and cells: (1) mouse corneal cells of the epithelial, stromal and endothelial layers; (2) corneas at 14 days after corneal transplantation with or without miR-122 overexpression (agomir-122, 20 *μ*M; Guangzhou RiboBio Co., Ltd, Guangzhou, China); (3) T cells (treated with plate-coated anti-CD3 (1 *μ*g/ml) and soluble anti-CD28 (1 *μ*g/ml) for 8 h), B cells, bone marrow-derived dendritic cells and bone marrow-derived macrophages (all treated with or without LPS (10 ng/ml) for 8 h); (4) mouse TKE2 cells treated with or without agomir-122 (20 *μ*M) for 24 h; (5) mouse keratocyte cells treated with or without antagmir-122 (20 *μ*M) (Guangzhou RiboBio Co., Ltd) for 24 h. The RNA concentrations were determined using UV spectrophotometry. Real-time quantitative PCR analysis was performed using specific primers for mouse *CPEB1 *(F, 5′- AGTTGCCAGCAGACTTCCAG-3′; R, 5′- AGGCACAAGCCATTTGCATC-3′) and miR-122 (MIMAT0000246, Guangzhou RiboBio Co., Ltd). U6 and GAPDH were used as the internal controls for miR-122 and CPEB1 detection, respectively.

### Western blotting

RIPA lysis buffer containing protease inhibitors was used to prepare total protein extracts from corneal grafts after PKP surgery or a mouse limbal epithelial stem cell line treated with or without agomir-122 (20 *μ*M) for 72 h. Samples were loaded onto 12% SDS-PAGE gels and subjected to electrophoresis. Proteins were transferred to nitrocellulose membranes and subsequently probed using an antibody for CPEB1 or GAPDH.

### Apoptosis analysis

A mouse corneal keratocyte cell line was transfected with agomir-122 (20 *μ*M), antagomir-122 (20 *μ*M), or a plasmid carrying CPEB1 cDNA. Alternatively, cells were transfected with a plasmid carrying CPEB1 cDNA with wild-type or miR-122-binding site-mutated 3′-UTR. The cells were then treated with or without a mixed cytokine cocktail containing 100 ng/ml TNF-*α*, IL-1*β* and TNF-*γ* (Peprotech, Rocky Hill, NJ, USA). After 48 h, the cells were stained with annexin V (Keygen Biotech, Nanjing, China) per the manufacturer’s instructions, and the percentage of apoptotic cells (annexin V+) was determined by flow cytometry.

### Treating mice with agomir-122 after corneal transplantation

The mouse allograft PKP model was established as mentioned above. The mice were treated locally in the eye with either 5 nmol agomir-native control or an equal amount of agomir-122 four times daily for 30 days. The survival conditions of the grafts in the two groups were observed daily for 30 days post-treatment. Animals with surgical complications were excluded from this study.

### Statistical analysis

The significance of differences in CPEB1 and miR-122 expression, luciferase activity and apoptosis rate was determined by Student’s *t*-test. The significance of difference in graft survival rates was determined by log-rank test.

## Figures and Tables

**Table 1:** The miRNAs that showed the largest correlation with the transplantation rejection

**MicroRNA**	**Fold change**	***P*****-value**
*Up*		
mmu-miR-714	2.213845	0.00717
mmu-miR-710	3.842705	0.029284
*Down*		
mmu-miR-5709	0.325458	0.021557
mmu-miR-3091-3p	0.340795	0.012828
mmu-miR-3110-3p	0.318219	0.024554
mmu-miR-1a-3p	0.325522	0.009227
mmu-miR-1694-5p	0.477831	0.041095
mmu-miR-122-5p	0.188281	0.033376
