# MicroRNA-122 ameliorates corneal allograft rejection through the downregulation of its target CPEB1

**DOI:** 10.1038/cddiscovery.2017.46

**Published:** 2017-08-21

**Authors:** Ting Wang, Fengjie Li, Wenwen Geng, Qingguo Ruan, Weiyun Shi

**Correction to:** *Cell Death Discovery* (2017) **3**, 17021; doi:10.1038/cddiscovery.2017.21; published online 15 May 2017

Since the publication of this paper, the authors realised that they had not included the funding information for the work. The necessary funding information is as follows:

This work was supported by the National Natural Science Foundation of China, Beijing, China (81470611, 81530027, and 81471554); the National Basic Research Program of China, Beijing, China (2013CB967004); the Taishan Scholar Program Phase II, Jinan, China (ts20150215); the Shandong Province Key Research and Development Program, Jinan, China (2015GGH318010); the Innovation Project of Shandong Academy of Medical Sciences; and the Shenzhen Municipal Science and Technology Innovation Committee (JCYJ20160531185449995).

The authors apologise for this error.

The html and pdf versions have been rectified, and now carry the corrected paper.

